# Construction of a mixed ligand MOF as “green catalyst” for the photocatalytic degradation of organic dye in aqueous media†

**DOI:** 10.1039/d1ra02994k

**Published:** 2021-07-06

**Authors:** Alamgir, Khalid Talha, Ying-Jie Wang, Raza Ullah, Bin Wang, Lu Wang, Wei Wu, Sha Chen, Lin-Hua Xie, Jian-Rong Li

**Affiliations:** Beijing Key Laboratory for Green Catalysis and Separation, Department of Environmental Chemical Engineering, Beijing University of Technology Beijing 100124 China xielinhua@bjut.edu.cn; Beijing Key Laboratory on Regional Air Pollution Control, Faculty of Environment and Life Sciences, Beijing University of Technology Beijing 100124 P. R. China

## Abstract

In the past few years, metal–organic frameworks (MOFs) have emerged as a class of fascinating materials for photocatalysis. Herein, a new MOF formulated as [Zn(bpe)(fdc)]·2DMF (BUT-206, bpe = 1,2-bis(4-pyridyl) ethylene, H_2_fdc = 2,5-furan dicarboxylic acid, DMF = *N*,*N*-dimethylformamide) is reported, which was synthesized under solvothermal conditions and applied for photocatalytic degradation of dyes (crystal violet and rhodamine B). Noteworthily, BUT-206 exhibited high photocatalytic activity toward the degradation of crystal violet without using any photosensitizer or cocatalyst under UV-irradiation. The photocatalytic degradation of crystal violet by BUT-206 was effective with a degradation efficiency of 92.5% within 120 minutes. The effects of key parameters including pH, amount of photocatalyst and initial concentration of dye on the dye degradation processes were examined, and the kinetics of dye degradation was established by the pseudo-first order kinetic equation. Furthermore, BUT-206 showed good cyclic stability in photocatalytic performance for up to five regeneration cycles, making it a potential green catalyst for dye degradation.

## Abstract 2

A new metal–organic framework named BUT-206 has been successfully synthesized, which shows a high photocatalytic activity toward the degradation of crystal violet without using any photosensitizer or cocatalyst under UV-irradiation.

## Graphical abstract

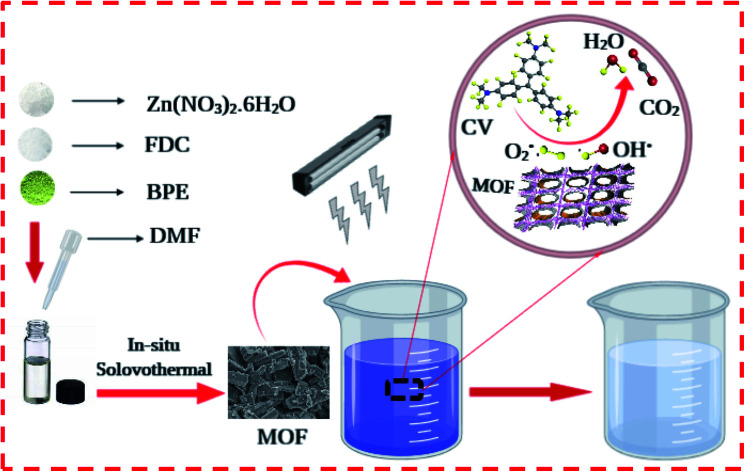

## 1. Introduction

With the fast technological development and the growth of industrialization, environmental protection, particularly water pollution remediation, has become one of the most critical issues in the present century faced by humans worldwide.^1,2^ A huge amount of industrial wastewater is produced in many industrial activities which often contains a variety of wastes including inorganic and organic compounds. The insufficient treatment of these wastes produces discharge of organic pollutants into surface water reservoirs, which poses a high risk to the purity of water systems and human health.^3,4^ In particular, organic dyes are usually highly toxic, not easily biodegraded, stable in water, and even potentially human mutagenic and carcinogenic.^5,6^ Therefore, to effectively remove organic dyes from wastewater, a series of conventional and advanced methods have been used, including ion exchange, coagulation/flocculation, adsorption, chemical oxidation and photocatalysis.^7–9^ Among them, photocatalysis, in which the *in situ* generation of highly reactive radical species (*e.g.* ˙OH and ˙O_2_^−^) for the conversion of organic dyes into CO_2_ and H_2_O, has been ascertained to be a promising method, and most importantly it is highly efficient and economical.^10–12^

Till now, different types of semiconductor photocatalysts, starting from metal oxides to metal salts and their composites have been used for dye degradation.^13,14^ However, these catalysts suffer from electron–hole pairs recoupling, low energy consumption efficiency, and easily clustering.^15^ With the growing concern about environmental remediation and human health, green technologies to degrade the contaminants in wastewater with high efficiency and low cost are in high demand.^16^ Particularly, high performance catalysts need to be developed to efficiently handle these contaminants.^17^

Metal–organic frameworks (MOFs), as considered to be a class of attractive and fascinating novel materials, constructed from organic ligands and metal ions, have publicized great potency for gas storage, separation, heterogeneous catalysis, sensing, proton conduction.^18–23,47,48^ In addition, some MOFs show semiconducting behavior under light, suggesting that they can be possibly used as photocatalysts.^24,25^ MOFs have many advantages, such as unsaturated metal sites and the catalytically active organic ligands as compared to traditional inorganic semiconductors.^14^ The terminated structural deficiencies of MOFs are the source of electron–hole recoupling and the electron–hole recombination.^26^ Alvaro *et al.* first demonstrated the degradation of phenol by a MOF-5 based photocatalyst in 2007.^27^ Liu *et al.* and Xia *et al.* later reported efficient MOF photocatalysts for the degradation of organic dyes.^28,29^ A heterogeneous photocatalyst for the reduction of aqueous solution of Cr^VI^ ions and the degradation of organic dyes, was reported by Zhao *et al.*^30^ Recently, Dong *et al.* reported a 2D Zn-MOF for the degradation of organic dyes in water.^10^ Though these researches have demonstrated the great of potency of MOFs for the photocatalytic degradation of environmental contaminants, the study on the photocatalytic properties of MOFs is still in early age. For example, most of the reported MOF-based photocatalysts showed high photocatalytic activity towards the degradation of organic dyes while using cocatalyst such as H_2_O_2_. However, using such cocatalyst in a large quantity during the decontamination of wastewater is not an environmental-friendly strategy. Therefore, it is in high demand to design a photocatalysts that can produce high photocatalytic efficiency in the absence of any photosensitizer or cocatalyst.^30^

Constructing MOFs with mixed ligands is a promising way to realize the desirable structure and property of MOFs. 1,2-Bis(4-pyridyl) ethylene is a rigid organic ligand, and its conjugated structure is helpful in electron transfer. Although a large number of MOFs based on this ligand have been reported, their photocatalytic activities have not been investigated.^31–34^ In this work, we report a new mixed-ligand MOF, [Zn(bpe)(fdc)]·2DMF (denoted as BUT-206, bpe = 1,2-bis(4-pyridyl) ethylene, H_2_fdc = 2,5-furan dicarboxylic acid, DMF = *N*,*N*-dimethylformamide), which was synthesized under solvothermal conditions. BUT-206 exhibited high photocatalytic activity towards the degradation of crystal violet without using any photosensitizer or cocatalyst. In addition, the photocatalytic activity of BUT-206 showed good stability up to five consecutive cycles. High photocatalytic activity in the absence of any photosensitizer or cocatalyst and high recyclability enable BUT-206 to be a potential green catalyst for the degradation of organic pollutants in environmental decontamination.

## 2. Experimental section

### 2.1 Materials and instruments

All general chemicals and solvents (AR grade) were commercially available and used as received. PXRD patterns were recorded on a BRUKER D8-Focus Bragg–Brentano X-ray powder diffractometer equipped with a Cu sealed tube (*λ* = 1.54178) at room temperature. Simulation of the PXRD pattern from the single-crystal structure was carried out by the diffraction crystal module of Mercury software. FT-IR data was recorded on SHIMADZU IR Affinity-1 instrument in the range of 200–800 nm. TGA data was obtained on a TGA-50 (SHIMADZU) thermogravimetric analyzer with a heating rate of 10 °C min^−1^ under nitrogen atmosphere. Gas adsorption isotherms were reported by a volumetric method using a Micromeritics ASAP2020 surface area and pore analyzer. UV-vis spectra and band gap were obtained with a UV-2600 spectrophotometer in the range of 200–800 nm at room temperature. Zeta potential (Pzc) of MOF samples at different pH values was measured on Malvern Zetasizer (Malvern Instruments Ltd, Malvern, UK). The morphology of MOF was examined through SU-3500 scanning electron microscope (SEM) and elemental distributions of MOFs structure were recorded through EDX analysis. Expected degradation products of crystal violet dye were analyzed by Gas chromatography-mass spectrometry (GC-MS) analysis. Photocurrent and Mott–Schottky measurements were carried out with a standard three-electrode system on a CHI 660E electrochemical work station (Chenhua Instrument, Shanghai, China). The Pt plate and typical Ag/AgCl were used as counter and reference electrode, respectively. The electrochemical impedance spectroscopy (EIS) was measured by Zennium electrochemistry work station with a bias potential of −0.6 V and over the frequency range of 10^−2^ to 10^5^ Hz.

### 2.2 Synthesis of MOF

Zn(NO_3_)_2_·6H_2_O (30 mg, 0.1 mmol), 2,5-furan dicarboxylic acid (H_2_fdc) (10 mg, 0.061 mmol), and 1,2-bis(4-pyridyl) ethylene (bpe) (6.5 mg, 0.036 mmol) were ultrasonically dissolved in 2 mL of DMF in a 5 mL Pyrex vial and sealed. The reaction system was then heated at 100 °C for 48 hours in an oven. After cooling to room temperature, the resulting block shaped crystals (yield: 80% based on the Zn) were collected by filtration, and washed with DMF and acetone.

### 2.3 Single-crystal X-ray diffraction

The X-ray diffraction data of as-synthesized BUT-206 were together by means of an Agilent Supernova CCD diffractometer (a mirror monochromator, Cu-Kα source, *λ* = 1.54184 Å). The datasets were amended by empirical absorption correction using spherical harmonics, implemented in the SCALE3 ABSPACK scaling algorithm. The structure was solved using direct methods and polished by full-matrix least-squares on *F*^2^ with anisotropic displacement using the SHELXTL software package.^35^ Hydrogen atoms of ligands were refined with isotropic displacement parameters. The disorderly solvents in holes might not be shaped in terms of atomic sites, but were cured by means of the MASK routine in the Olex2 software package.^36^ The crystal parameters and structure refinement of BUT-206 were summarized in Table S1 (for details, see CCDC 1982845).†

### 2.4 Photo-catalytic degradation experiments

This experiment was conducted in aqueous media in order to check the photo-catalytic degradation activity of crystal violet dye at room temperature. For this purpose, conditions were optimized. The solution of 100 mL was added to 250 mL glass beaker in which 0.5 g L^−1^ catalyst dose was added. The concentration of solution was 5 ppm, while the pH = 6. Time taken for experiment was 120 minutes at 25 °C. Aliquant part of used sample was withdrawn for analysis at veritable time interval, filtered to eliminate the undissolved particles and then studied by UV-vis spectrophotometer, in which high pressure UV-Hg source (80 W) was used as light source. To confirm the accuracy in result, experimentation was performed in replicate in order to obtained average results. After completion of single cycle, the used MOF-based catalyst was recycled by filtration, water-washed and followed by acetone, dried and then studies their photo-constancy. The percentages, (*X*%) of the crystal violet dye degradation were measured by applying the eqn (1), where *C*_0_ = initial concentration of the crystal violet dye and *C* = concentration at specified time interval.1
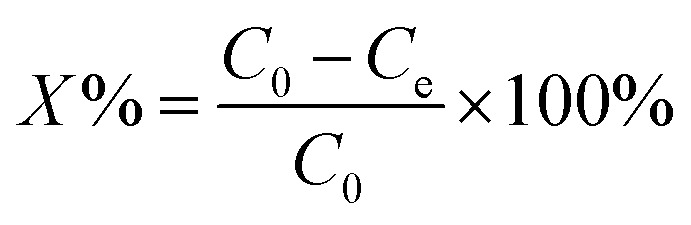


## 3. Results and discussion

### 3.1 Single-crystal structure

**Fig. 1:**
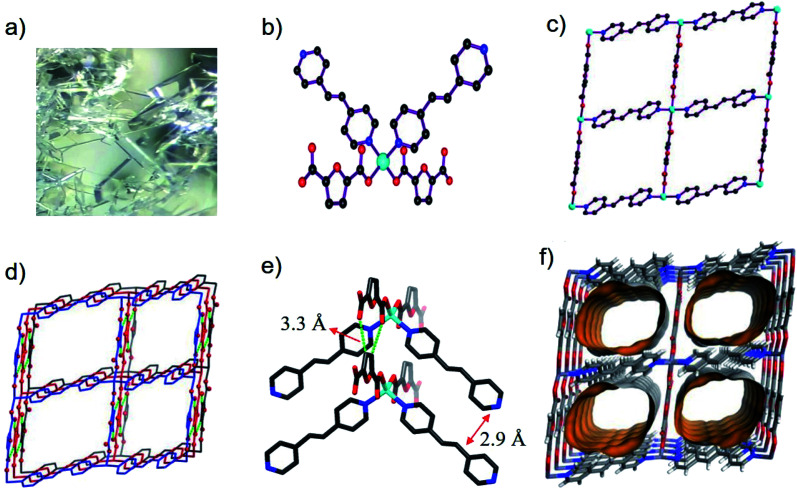
(a) Optical photo of single crystals of BUT-206; (b) the coordination environment of Zn^2+^, (c) a 2D layer, (d) stacking of such 2D layers, (e) weak interactions between neighboring 2D layers, and (f) the channels in BUT-206.

Single-crystal X-ray diffraction data showed that BUT-206 crystallizes in the monoclinic space group *P*2_1_/*c* with a formula of [Zn(bpe)(fdc)]·2DMF. In the asymmetric unit of BUT-206, there are halves of Zn^2+^ ion, bpe and fdc^2−^ ligands, and one guest DMF molecule. Each Zn^2+^ ion is coordinated with two carboxylate O atoms from two fdc^2−^ ligands and two pyridine N atoms from two bpe ligands in a tetrahedral geometry with the Zn–O and Zn–N bond lengths ranging from 1.988(3) Å to 2.053(4) Å (Fig. 1a), and the other two carboxylate O atoms of fdc^2−^ ligands are weakly bonded with the Zn^2+^ ions with a Zn⋯O distance of 2.67 Å. Each ligand (bpe or fdc^2−^) is bridging two Zn^2+^ ions. The alternate connection of the Zn^2+^ ions and the ligands leads to a 2D crumpled 4^4^ layer (sql net) (Fig. 1b). The stacking of such 2D layers along the *b* axis results in the 3D structure of BUT-206 (Fig. 1c). The stacking of these 2D layers is stabilized by the π–π interactions between the bpe ligands (interplane distance: 2.9 Å) and C–H⋯O non-classical hydrogen bonding interactions (C⋯O distance: 3.32 Å) between the fdc^2−^ ligands on neighboring layers. There are 1D rectangular channels in size of ∼9 × 6 Å along the *b* axis which are occupied by guest molecules (two DMF molecules per formula) (Fig. 1d). The total potential solvent area volume is 43.4% of the crystal volume as estimated by Platon.

### 3.2 TGA, PXRD and N_2_ sorption

**Fig. 2:**
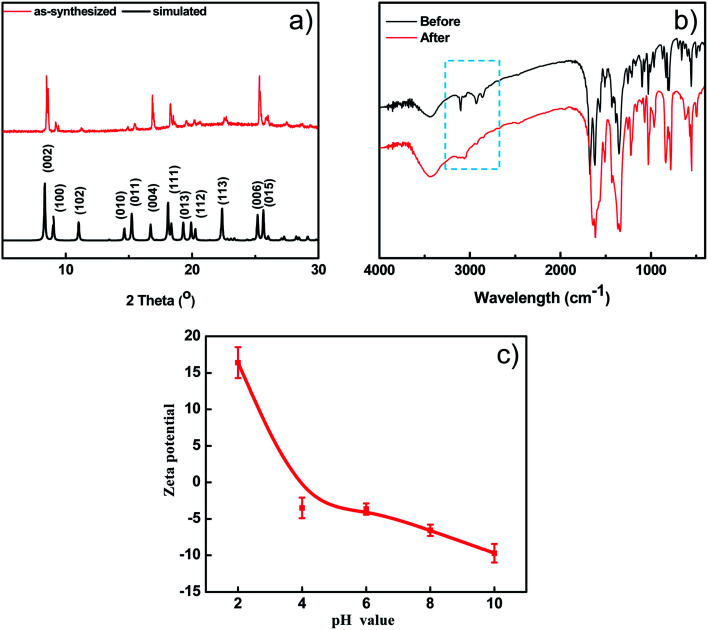
(a) The simulated PXRD pattern of BUT-206 and PXRD pattern of as-synthesized sample of BUT-206; (b) the FT-IR spectra of BUT-206 before and after photocatalytic reaction; (c) the pH_pzc_ of BUT-206.

The thermal stability of BUT-206 was accessed by a thermalgravimetric analyzer in a temperature range of 25 to 800 °C with a heating rate of 10 min^−1^ under nitrogen atmosphere. TGA curve showed that the guest molecules were removed before 220 °C, and framework started to decompose at around 300 °C as depicted in Fig. S1b.† The PXRD pattern of the as-synthesized sample of BUT-206 well matches the simulated one obtained from its single-crystal data (Fig. 2a), indicating pure phase of the sample. After water treatment at room temperature for 1 week, the PXRD pattern of the BUT-206 sample significantly changed (Fig. S6†), indicating a severe structural transformation. Attempt to determine the structure of the sample after structural transformation failed. N_2_ adsorption measurement at 77 K was performed for BUT-206, before the adsorption experiment the sample was activated by acetone as shown in Fig. S1(a),† only surface adsorption was observed, suggesting the porous framework structure of BUT-206 may collapse after removal of the guest molecules.

### 3.3 FT-IR spectra

In the FT-IR spectrum of BUT-206, the peaks at 1620–1680 cm^−1^ are attributed to the stretching of C=C bonds of bpe ligands. The peaks at 1510–1650 cm^−1^ are corresponding to the asymmetrical vibration of carboxylate groups of fdc^2−^ ligands. The strong absorption at 1661 cm^−1^ resulting from the amide bonds of DMF molecules confirms the existence of the guest molecules. The peaks at 2800–3000 cm^−1^ are due to the stretching of C–H bonds in BUT-206, as shown in Fig. 2b.^37^

### 3.4 pH_pzc_ and band gap analysis

The surface charge property of the BUT-206 in the aqueous suspension was determined in the form of zeta potential. The zeta potential of BUT-206 as a function of equilibrium pH of the suspension in the range of 2–10 was determined. The results showed that the surface of BUT-206 was negatively charged at pH = 6, 8 and 10, and its surface became positively charged when pH = 2 and 4. The pH values of point of zero charge (pH_pzc_) for BUT-206 was about 4.9, as shown in Fig. 2c. The pH_pzc_ is a concept relating to the adsorption phenomena and is defined as the pH at which the surface of the catalyst is uncharged. At pH values higher than the pH_pzc_ the catalyst surface is negatively charged and thus the adsorption of cation is favored, while at pH values lower than the pH_pzc_ the surface of catalyst is positively charged and hence the adsorption of anions is favored.

To explore the band gap fluctuation, UV-vis spectrum of BUT-206 was recorded. BaSO_4_ was used as a reference standard. Band gap of BUT-206 was determined by applying Tauc relation (*Ahν*)^*n*^ = *C*(*hν* − *E*_g_), where *A* is absorbance, *h* is the Planck's constant, *ν* is frequency and *E*_g_ is the band gap energy. The *n* has values of 2 for the allowed direct and 1/2 for the allowed indirect transitions. Therefore, (*Ahν*)^*n*^ (*y* axis) was drawn *versus hν* (*x* axis), which gave the band gap (*E*_g_) value indicating its semiconducting property. Finally, the direct and indirect band gaps values for BUT-206 were calculated to be 3.35 and 2.74 eV, respectively, as shown in Fig. S3a and b.†

### 3.5 Photo-catalytic degradation

**Fig. 3:**
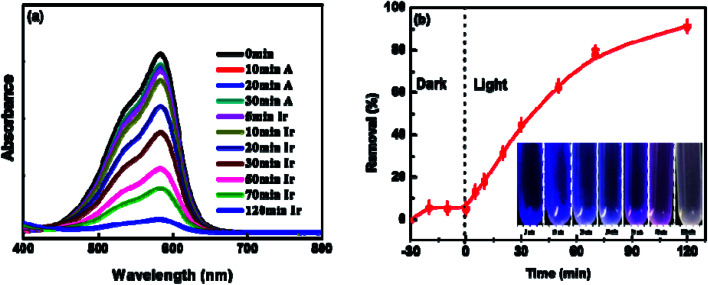
(a) Absorption spectrum of crystal violet at different intervals of time; (b) the degradation of crystal violet under optimized conditions: catalyst dose = 0.5 g L^−1^, pH = 6, concentration of crystal violet = 5 ppm, time = 120 minutes and temp. = 25 °C.

To explore the photocatalytic activity of BUT-206 for the degradation of organic dyes in water, we selected two commonly used organic cationic dyes (crystal violet and rhodamine B) as target pollutants. The effect of UV-light on the photo-degradation of crystal violet and rhodamine B dyes were first tested by carrying out control experiments under UV-irradiation in the absence of MOF. Entirely 3% degradation of both the dyes was observed within 120 minutes of UV-irradiation (Fig. S4a†). For photo-catalytic degradation experiments with the presence of MOF, dye solutions containing the MOFs were agitated continuously for 30 minutes in dark to reach the adsorption equilibrium. The amount of crystal violet adsorbed on the MOF sample was found to be 5% was depicted in Fig. S4b.† Their distinctive absorption peaks appearing at 589 nm and 554 nm were monitored to check the photocatalytic degradation efficiency of BUT-206 towards crystal violet and rhodamine B. The degradation of crystal violet and rhodamine B were ascertained for 120 minutes under UV-light irradiation, the liquid samples were withdrawn after different intervals of time from the 5^th^ to 120^th^ minutes. The variations in the absorption intensity and concentrations of crystal violet and rhodamine B solutions *C*_*t*_/*C*_0_ with the irradiation time were plotted. The photocatalytic degradation efficiency of BUT-206 toward crystal violet was 92.5% while the degradation of rhodamine B was negligible at the same conditions, as shown in Fig. 3 and Fig. S5,† respectively. The tendency of degradation observed for rhodamine B over BUT-206 suggested that there might be some factors which are responsible for the non-quenching of rhodamine B. The factors that affect the degradation process could be surface charges of photocatalyst, size of the dye, pH of the solution and concentration of dye plays an essential role in deciding the potency of interaction between photocatalyst and dye molecule under analysis, in this way completely affecting the degradation process. Absorption spectra of crystal violet dye at different intervals of time indicated that BUT-206 could be used as a selective catalyst for the photocatalytic degradation of crystal violet in the absence of cocatalyst or photosensitizer as shown in Fig. 3a and b. Hence, the effect of several factors on the catalytic performance of BUT-206 was systematically studied.

### 3.6 Effect of pH and dosage of the catalyst

**Fig. 4:**
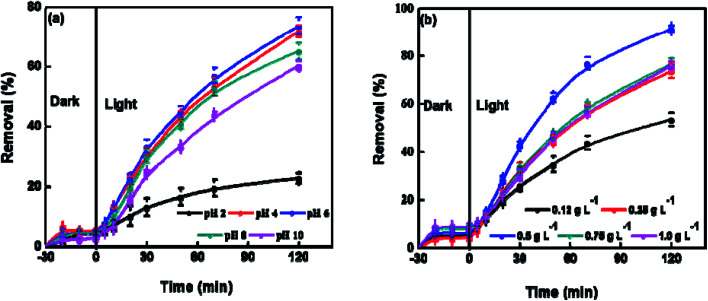
Experimental parameters investigation for the photocatalytic degradation of crystal violet with BUT-206: (a) effect of pHs (2–10) on the photocatalytic degradation performance; (b) effect of dose of MOF (0.12–1.0 g L^−1^) on the photocatalytic degradation performance.

Variations in the pH of electrolyte could alter the surface charge of photocatalyst and displace the routes of some redox reactions. The pH also disturbs the adsorption of substrates on the surface of photocatalyst, and thus affects the reactivity and reaction rate. The effect of pH on the photocatalysis performance of BUT-206 was studied at room temperature. The degradation efficiency of crystal violet increased by raising the pH of solution from 4 to 6 and decreased beyond this limit (Fig. 4a). In strong acidic condition, the inorganic radicals ClO˙ can be produced *via* the reaction of Cl^−^ ions with hydroxyl radicals. These inorganic radicals express a much lower reactivity than ˙OH, so the degradation efficiency of crystal violet decreased at pH = 2. Similarly, at lower pH a tough competition between crystal violet and Cl^−^ anions with respect to ˙OH starts, which decreases the degradation efficiency of crystal violet. Therefore, it is desirable to increase the pH of electrolyte for the enhancement of degradation efficiency. The charges on catalyst surface become positive and negative at pHs lower and higher than zeta potential, respectively. In the strong acidic condition with pH = 2, the repulsive force between positively charged catalyst surface and protonated crystal violet molecules prevent the approaching of crystal violet molecules to the catalyst surface, where hydroxyl radicals formed. With the rising of pH, this hurdle decreased and the maximum degradation efficiency achieved at pH = 6 as shown in Fig. 4a.^38^

The photocatalyst dose also affects the preliminary rate of photocatalytic degradation of contaminants. Therefore, the effect of the amount of BUT-206 on the degradation of crystal violet was also examined in a succession of solutions by changing the amount of MOF catalyst (0.12, 0.25, 0.5, 0.75 and 1.0 g L^−1^, respectively) and the results are shown in Fig. 4b. It can be seen that with the increase in the dose of MOF catalyst from 0.12 to 0.5 g L^−1^ the degradation efficiency increases and then decreases when using relatively higher doses from 0.75 to 1.0 g L^−1^. With the high concentration of catalyst, the non-transparency of suspension, light scattering phenomenon and accumulation of solid particles increases. Consequently, masking effect of excess particles happens which cover some parts of the photosensitive surface. Hereafter, the diffusion penetration of the photons reduces and fewer catalyst particles can be initiated. Consequently, the production of hydroxyl radicals and the degradation efficiency decreases. The optimum degradation rate was ascertained in the presence of 0.5 g L^−1^ catalyst and this optimized value was used in the succeeding studies.^39,40^

### 3.7 Effect of initial concentration of dye

**Fig. 5:**
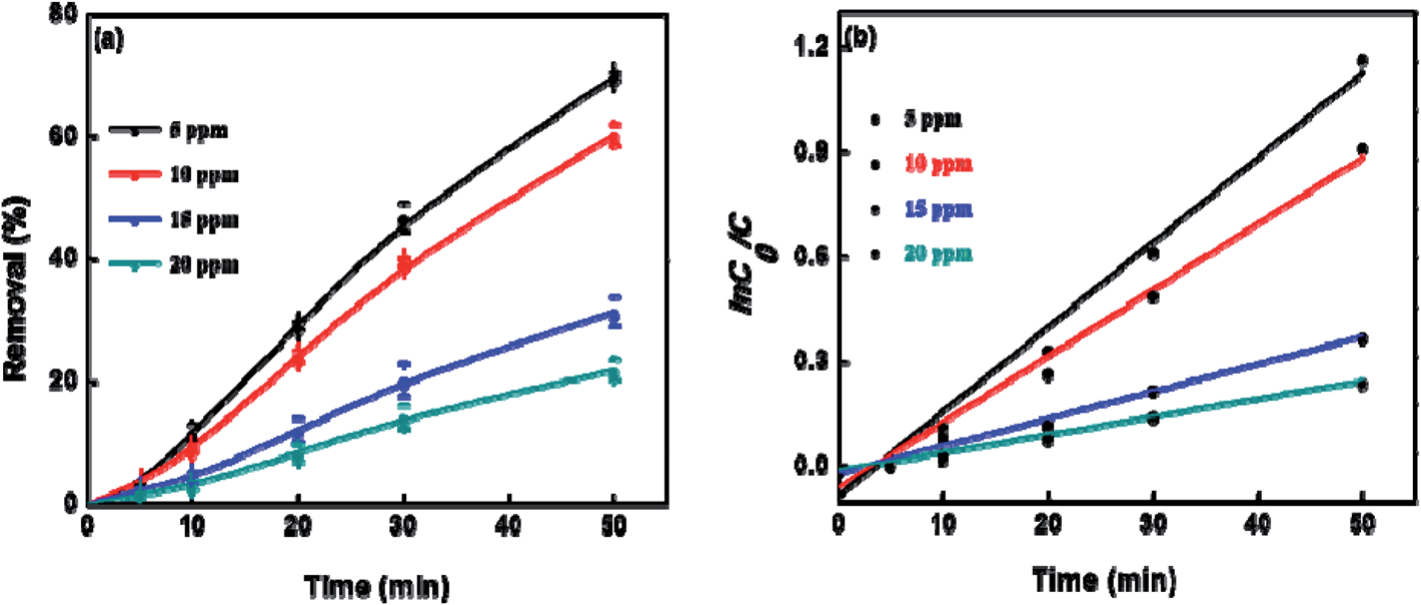
(a) Photocatalytic degradation of crystal violet at different initial concentrations; (b) pseudo-first order degradation kinetics using different initial concentration of crystal violet; experimental conditions: pH = 6, dosage of catalyst = 0.5 g L^−1^, time = 50 minutes and temp. = 25 °C.

To explore the effect of initial concentration of crystal violet on the photocatalytic performance of BUT-206, four different concentrations of the dye solutions, namely 5, 10, 15, and 20 ppm were used for the control experiments. The results showed that initial concentration of crystal violet had an obvious influence on the photocatalytic degradation rate (Fig. 5a). The degradation rate using BUT-206 as catalyst declines with the rising of initial concentration of crystal violet. It might be owing to the reason that only a certain amount of ˙OH radicals could be generated with a certain quantity of photocatalyst and UV-light, which then reacted with the different initial concentrations of crystal violet. Additionally, the opaqueness of solution rises with the increasing of dye concentration and diffusion of UV-light over the solution turns hard, generating comparatively a smaller amount of the extremely reactive ˙OH radicals.^41^ To investigate the kinetics of the photo-catalytic degradation process, the values of ln(*C*_0_/*C*) *versus* irradiation time was plotted using different initial concentrations of the crystal violet. The degradation processes can be well modelled by the pseudo-first order kinetic equation as given in eqn (2), where *C*_0_ is the initial concentration of crystal violet and *C* is the concentration at time *t*, while *k*_app_ is the apparent rate constant and *t* is the time. A linear relationship can be obtained between ln(*C*_0_/*C*) *vs.* irradiation time for different initial concentrations of crystal violet dye solutions as shown in Fig. 5b. The rate constant values *k*_app_ (min^−1^) were estimated from the linear fitting of the plot with the pseudo-first order kinetic equation, and the attained results are listed in Table 1. Obviously, the rate constant values decrease as the increasing of the initial concentrations of crystal violet dye solutions.2
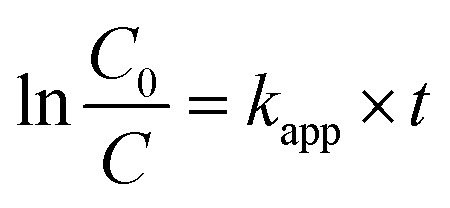


**Table 1:** Observed rate constants and degradation efficiency (%) at different initial concentrations of crystal violet

Conc. of crystal violet (ppm)	Degradation efficiency (%)	*k*_app_ (min^−1^)	*R* ^2^
5	69.9	0.024	0.983
10	60.5	0.019	0.984
15	31.6	0.008	0.991
20	22.0	0.005	0.991

### 3.8 Mechanism of photocatalytic activity

The possible mechanism for the photocatalytic degradation of crystal violet by BUT-206 is proposed as follows. Once UV-light strikes the aqueous solutions of crystal violet containing BUT-206 as photocatalyst, the electrons in the valance band of BUT-206 are excited and absorb photons of energy and moves to the conduction band. These photons of energy are equal to or larger than the band gap of the semi-conductor, and after absorbing these photons by conduction band, positive holes in the valance band are formed. The excited electrons can either recoupling with positive holes or react with dissolved O_2_ molecules to form reactive superoxide anion radical (O_2_˙^−^). Correspondingly, water molecules can be oxidized by positive charged holes which are adsorbed on the surface of catalyst to form extremely active hydroxyl radical (˙OH). These produced ˙OH and O_2_˙^−^ bearing high oxidation potency and therefore stimulate the degradation of organic pollutants. These outcomes prove that both the radicals O_2_˙^−^ and ˙OH contributed to the photocatalysis processes, but the processes were mostly depended on the hydroxyl radicals. Consequently, the following route may show the potential photocatalytic degradation mechanism of crystal violet in water by the photocatalyst BUT-206 under UV-irradiation.

3 MOF + *hν* → e^−^ + h^+^ + MOF

The positive holes h^+^ (charge carrying body in semiconducting materials) combining with H_2_O and the excited electrons combining with O_2_ on the surface of MOF can be shown as follows:

4 h^+^ + H_2_O → OH˙ + H^+^

5 e^−^ + O_2_ → O_2_˙^−^

The highly reactive OH˙ and O_2_˙^−^ oxidize the crystal violet molecules (C_25_H_30_ClN_3_), which are adsorbed on the surface of the BUT-206, producing CO_2_, H_2_O, NO_3_^−^ and some other comparatively less toxic products.^42–44^ The molecular structures of these expected minor degradation products of crystal violet dye are listed in Table S2.†^45,46^ To confirm the formation of OH˙ and O_2_˙^−^ radicals, ESR spectra were recorded for the solution containing BUT-206 under full light irradiation. As shown in Fig. S8,† typical signal peaks for the hydroxyl radicals and superoxide anion radicals were observed, although the signal intensities are relatively low, suggesting that these radicals were formed in the photocatalytic reactions with BUT-206 as catalyst. To explore the charge transfer efficiency of BUT-206, the photocurrent, electrochemical impedance spectra (EIS), and Mott–Schottky measurements were also carried out (Fig. S9†).

6 C_25_H_30_ClN_3_ + (OH˙, O_2_˙^−^) → CO_2_ + H_2_O + NO_3_^−^ + other simple fragments

### 3.9 Recyclability

**Fig. 6:**
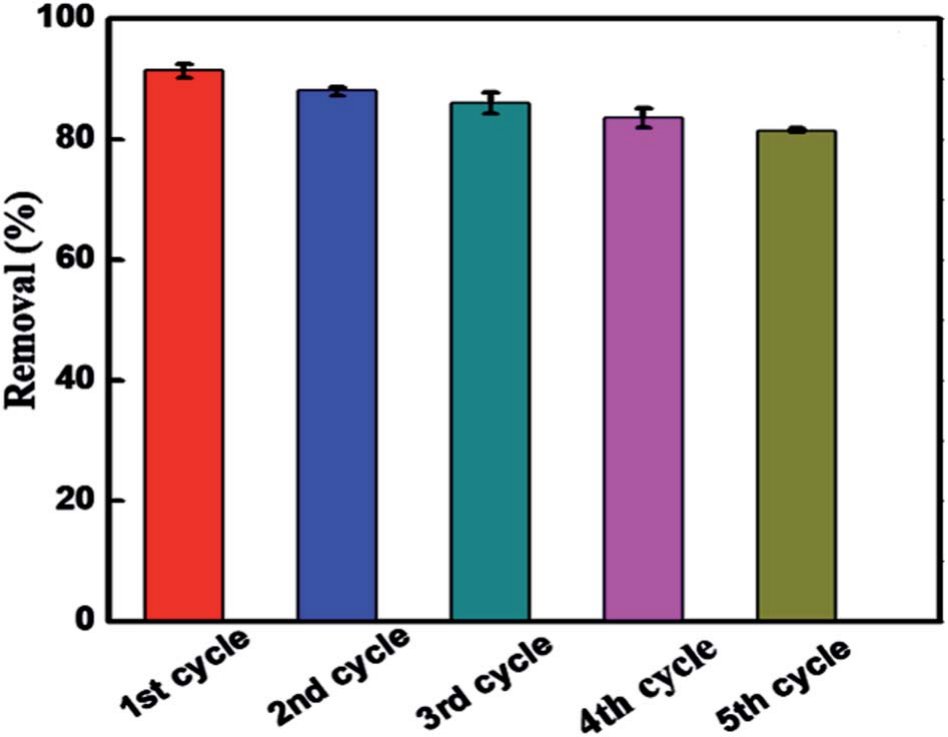
Reproducibility of BUT-206 for the photocatalytic degradation of crystal violet up to five cycles.

The recyclability of the BUT-206 photocatalyst is an important parameter for the economical and green water treatment. To explore the feasibility of BUT-206, its recyclability was examined over five consecutive cycles. After each cycle, the sample was restored by centrifugation and washed consecutively for three times with deionized water followed by acetone, and ethanol. The complete process was as follows: the regenerated sample was immersed into 5 mL of solvent in a vial and sonicated for a while, and then filtered. The restored sample was dried at high vacuum overnight, and then use again for the next cycle. The MOF sample was used over five consecutive cycles to examine their photocatalysis performance, and the results are shown in Fig. 6. The degradation efficiency declined slightly after five consecutive cycles, which might be due to the irreversible adsorption of contaminant molecules on active sites of the catalyst. The PXRD pattern and SEM image were also recorded for the MOF sample after 5th regeneration cycles. As shown in Fig. S6 and S7,† the results suggested that the structure of the MOF changed during the reactions, although the photocatalytic activity largely retained.

## 4. Conclusion

In summary, we synthesized a new MOF denoted as BUT-206 by reacting zinc salt with a dicarboxylate ligand fdc^2−^ and a neutral ligand bpe. The structural architecture of MOF suggested that “one-pot” technique is a controllable method to design and synthesize functional mixed ligand MOF materials for selective photocatalytic applications. It has been established that BUT-206 is a highly efficient photocatalyst for the degradation of an aqueous solution of dye (crystal violet) without using any photosensitizer or cocatalyst. In addition, the MOF showed a good regeneration performance up to five cycles. This unique property suggested that BUT-206 is an economical and green potential candidate for wastewater treatment. It is predicted that more effective MOF-based photocatalysts can be synthesized toward the objective of green environmental restitution and photocatalytic degradation of dye-containing wastewater can be transmitted by using MOF materials as environmentally-sound and energy efficient photocatalysts.

## Conflicts of interest

There are no conflicts to declare.

## Supplementary Material

RA-011-D1RA02994K-s001

RA-011-D1RA02994K-s002

## References

[cit1] Wu Z., Yuan X., Zhang J., Wang H., Jiang L., Zeng G. (2017). ChemCatChem.

[cit2] Liu J., Diamond J. (2005). Nature.

[cit3] Sharma V. K., Feng M. (2019). J. Hazard. Mater..

[cit4] Zhang T., Lin W. (2014). Chem. Soc. Rev..

[cit5] Bedia J., Muelas-Ramos V., Peñas-Garzón M., Gómez-Avilés A., Rodríguez J. J., Belver C. (2019). Catalysts.

[cit6] Wang C.-C., Li J.-R., Lv X.-L., Zhang Y.-Q., Guo G. (2014). Energy Environ. Sci..

[cit7] Velegraki G., Miao J., Drivas C., Liu B., Kennou S., Armatas G. S. (2018). Appl. Catal., B.

[cit8] Verma A. K., Dash R. R., Bhunia P. (2012). J. Environ. Manage..

[cit9] Cheng Y.-J., Wang R., Wang S., Xi X.-J., Ma L.-F., Zang S.-Q. (2018). Chem. Commun..

[cit10] Dong J.-P., Shi Z.-Z., Li B., Wang L.-Y. (2019). Dalton Trans..

[cit11] Zhang M.-W., Lin K.-Y. A., Huang C.-F., Tong S. (2019). Sep. Purif. Technol..

[cit12] Xiang Q., Yu J., Jaroniec M. (2012). Chem. Soc. Rev..

[cit13] Zong Z.-a., Fan C.-b., Zhang X., Meng X.-m., Jin F., Fan Y.-h. (2019). Microporous Mesoporous Mater..

[cit14] Zhang M., Wang L., Zeng T., Shang Q., Zhou H., Pan Z., Cheng Q. (2018). Dalton Trans..

[cit15] Zeng T., Wang L., Feng L., Xu H., Cheng Q., Pan Z. (2019). Dalton Trans..

[cit16] Li J., Wang X., Zhao G., Chen C., Chai Z., Alsaedi A., Hayat T., Wang X. (2018). Chem. Soc. Rev..

[cit17] Liu X., Liu B., Li G., Liu Y. (2018). J. Mater. Chem. A.

[cit18] Burtch N. C., Jasuja H., Walton K. S. (2014). Chem. Rev..

[cit19] Yaghi O. M. and Millward A. R., US Pat., US7799120B2, 2010

[cit20] Yamada T., Otsubo K., Makiura R., Kitagawa H. (2013). Chem. Soc. Rev..

[cit21] Xie Z., Ma L., deKrafft K. E., Jin A., Lin W. (2010). J. Am. Chem. Soc..

[cit22] Li J.-R., Kuppler R. J., Zhou H.-C. (2009). Chem. Soc. Rev..

[cit23] Talha K., He T., Xie L.-H., Wang B., Zhao M.-J., Zhang Y.-Z., Chen Q., Li J.-R. (2020). New J. Chem..

[cit24] Silva C. G., Corma A., García H. (2010). J. Mater. Chem..

[cit25] Habisreutinger S. N., Schmidt-Mende L., Stolarczyk J. K. (2013). Angew. Chem., Int. Ed..

[cit26] Xiao J.-D., Jiang H.-L. (2018). Acc. Chem. Res..

[cit27] Alvaro M., Carbonell E., Ferrer B., Llabrés i Xamena F. X., Garcia H. (2007). Chem.–Eur. J..

[cit28] Liu C. X., Zhang W. H., Wang N., Guo P., Muhler M., Wang Y., Lin S., Chen Z., Yang G. (2018). Chem.–Eur. J..

[cit29] Xia Q., Yu X., Zhao H., Wang S., Wang H., Guo Z., Xing H. (2017). Cryst. Growth Des..

[cit30] Zhao H., Xia Q., Xing H., Chen D., Wang H. (2017). ACS Sustainable Chem. Eng..

[cit31] Xiong G., Wang Y., Sun Y., You L., Ren B., Xu Z., He Y., Ruhlmann L., Ding F. (2018). Eur. J. Inorg. Chem..

[cit32] Bisht K. K., Rachuri Y., Parmar B., Suresh E. (2014). J. Solid State Chem..

[cit33] Chen D.-M., Xu N., Qiu X.-H., Cheng P. (2015). Cryst. Growth Des..

[cit34] Liu Y., Chen D., Li X., Yu Z., Xia Q., Liang D., Xing H. (2016). Green Chem..

[cit35] Eubank J. F., Wojtas L., Hight M. R., Bousquet T., Kravtsov V. C., Eddaoudi M. (2011). J. Am. Chem. Soc..

[cit36] Sheldrick G. , SADABS, Program for scaling and correction of area detector data, University of Göttingen, Germany, 1997

[cit37] Dolomanov O. V., Blake A. J., Champness N. R., Schröder M. (2003). J. Appl. Crystallogr..

[cit38] Singh D., Nagaraja C. (2014). Dalton Trans..

[cit39] Talha K., Wang B., Liu J.-H., Ullah R., Feng F., Yu J., Chen S., Li J.-R. (2020). J. Environ. Chem. Eng..

[cit40] Ai L., Zhang C., Li L., Jiang J. (2014). Appl. Catal., B.

[cit41] Ullah R., Liu C., Panezai H., Gul A., Sun J., Wu X. (2020). Arabian J. Chem..

[cit42] Pan Y., Liu W., Liu D., Ding Q., Liu J., Xu H., Trivedi M., Kumar A. (2019). Inorg. Chem. Commun..

[cit43] Nezamzadeh-Ejhieh A., Shirzadi A. (2014). Chemosphere.

[cit44] Liao Y.-H. B., Wang J. X., Lin J.-S., Chung W.-H., Lin W.-Y., Chen C.-C. (2011). Catal. Today.

[cit45] Ullah R., Sun J., Gul A., Bai S. (2020). J. Environ. Chem. Eng..

[cit46] Parshetti G., Parshetti S., Telke A., Kalyani D., Doong R., Govindwar S. P. (2011). J. Environ. Sci..

[cit47] He T., Huang Z., Yuan S., Lv X.-L., Kong X.-J, Zou X., Zhou H.-C., Li J.-R. (2020). J. Am. Chem. Soc..

[cit48] Xie L.-H., Liu X.-M., He T., Li J.-R. (2018). Chem.

