# Supplementary material for: Construction of a mixed ligand MOF as “green catalyst” for the photocatalytic degradation of organic dye in aqueous media
Source: RSC Adv. 2021 Jul 6;11(38):23838–45. doi: 10.1039/d1ra02994k (PMC9036557; doi:10.1039/d1ra02994k)
Supplement: RA-011-D1RA02994K-s001 [file RA-011-D1RA02994K-s001.pdf]

Electronic Supplementary Material (ESI) for RSC Advances.
This journal is © The Royal Society of Chemistry 2021

Supporting Information

Construction of a mixed ligand MOF as "green catalyst" for the photocatalytic degradation of organic dye in aqueous media

Alamgir[a,§], Khalid Talha[a,§], Ying-Jie Wang, Raza Ullah[a], Bin Wang[a], Lu Wang[a], Wei Wu[a], Sha Chen[b], Lin-Hua Xie[a*], Jian-Rong Li[a]

[a]*Beijing Key Laboratory for Green Catalysis and Separation and Department of Environmental Chemical Engineering, Beijing University of Technology, Beijing 100124, China*

[b]*Beijing Key Laboratory on Regional Air Pollution Control, Faculty of Environment and Life Sciences, Beijing University of Technology, Beijing 100124, P. R. China*

E-mail: xielinhua@bjut.edu.cn

§ Alamgir and K. Talha contributed equally.

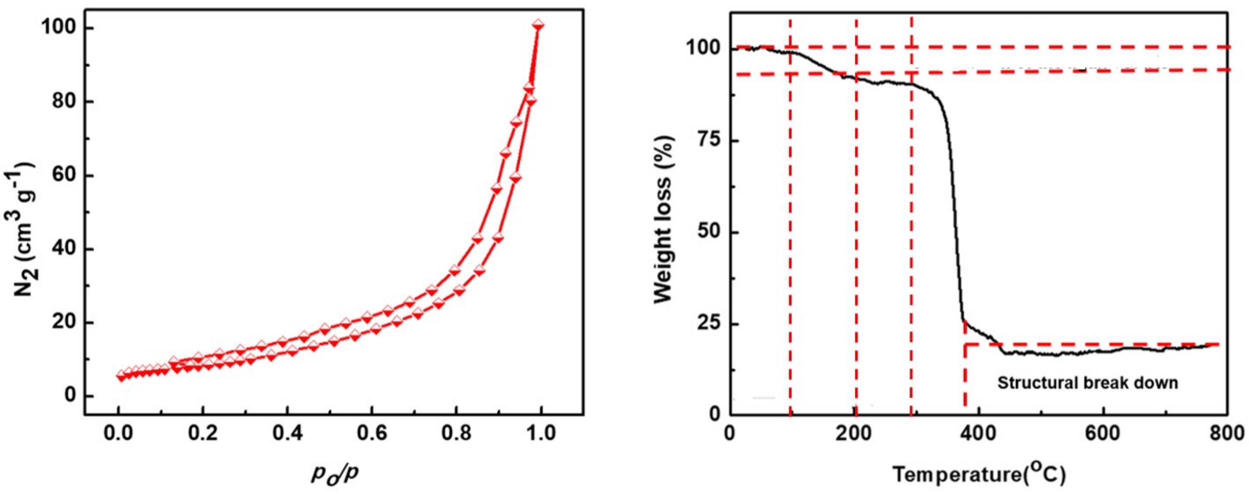

Fig. S1 (a) N_2 adsorption isotherm of **BUT-206** recorded at 77 K; (b) TGA spectra of **BUT-206.°**

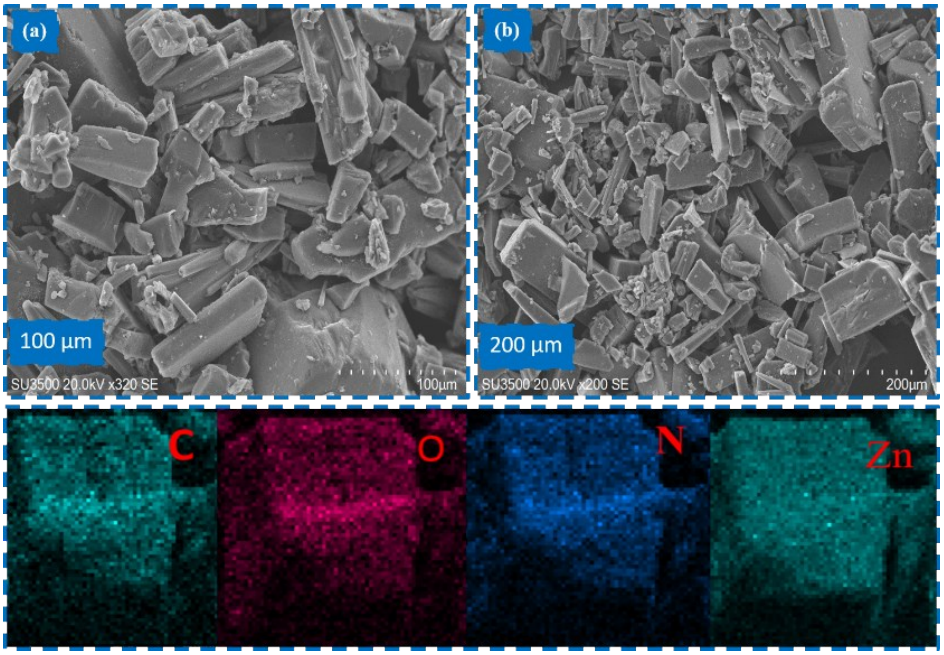

Fig. S2 SEM and EDAX mapping of **BUT-206**

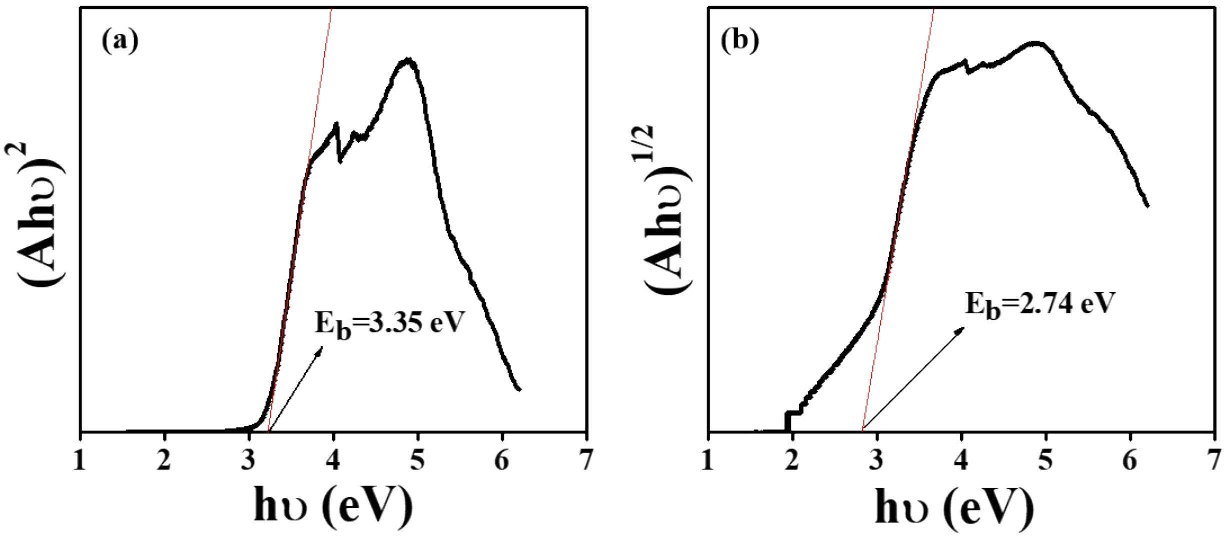

Fig. S3 The direct (a) and indirect (b) band gap values of **BUT-206**, respectively.

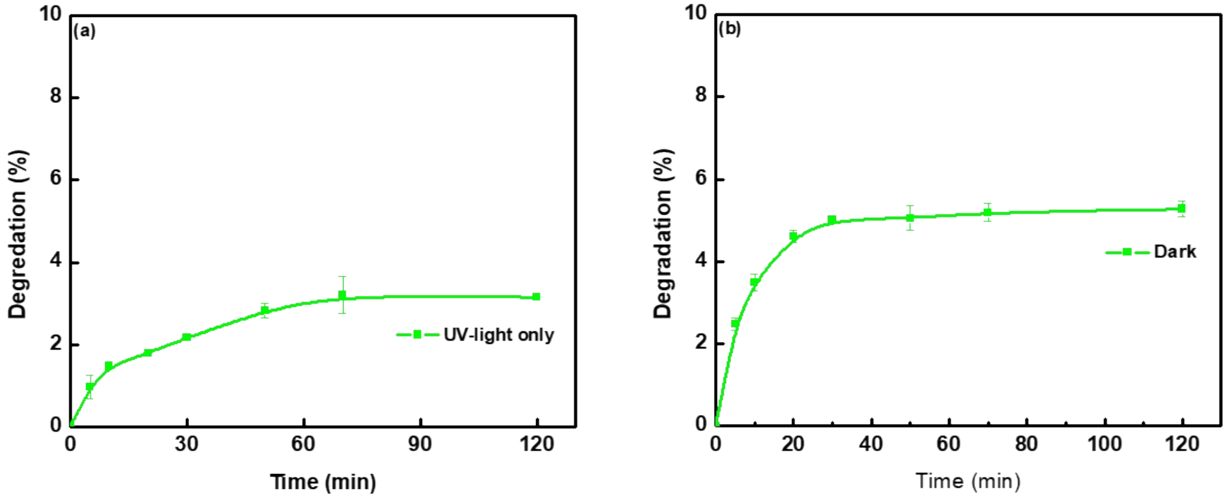

Fig. S4 (a) The degradation of crystal violet under UV-light in the absence of MOF; (b) adsorption of crystal violet by the MOF in dark condition.

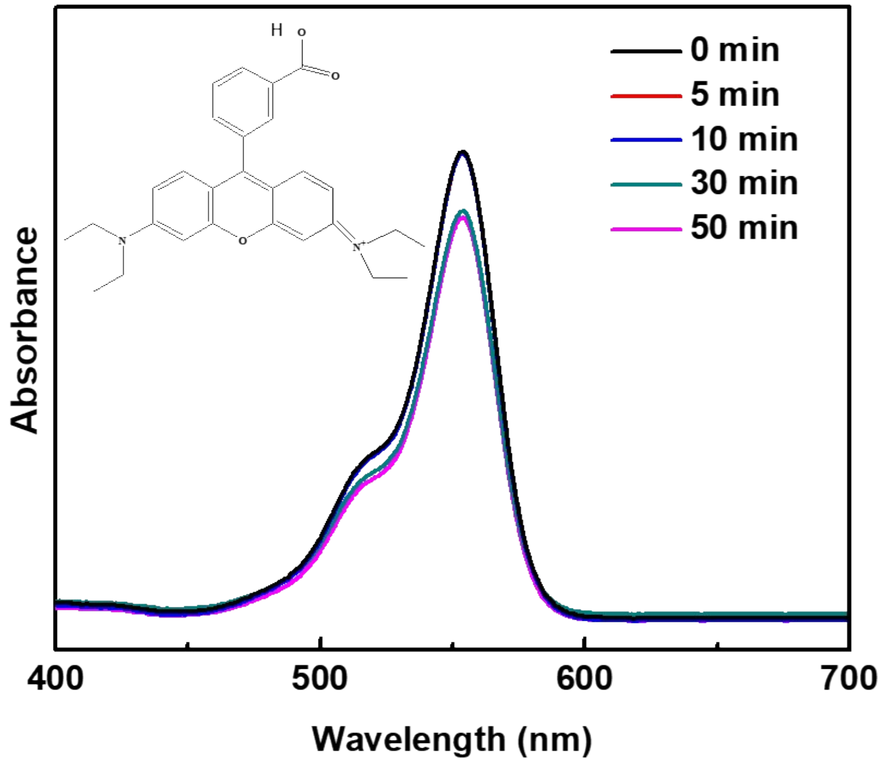

Fig. S5 Absorption spectra of rhodamine B at different intervals of time during the photocatalytic experiment with MOF as catalyst.

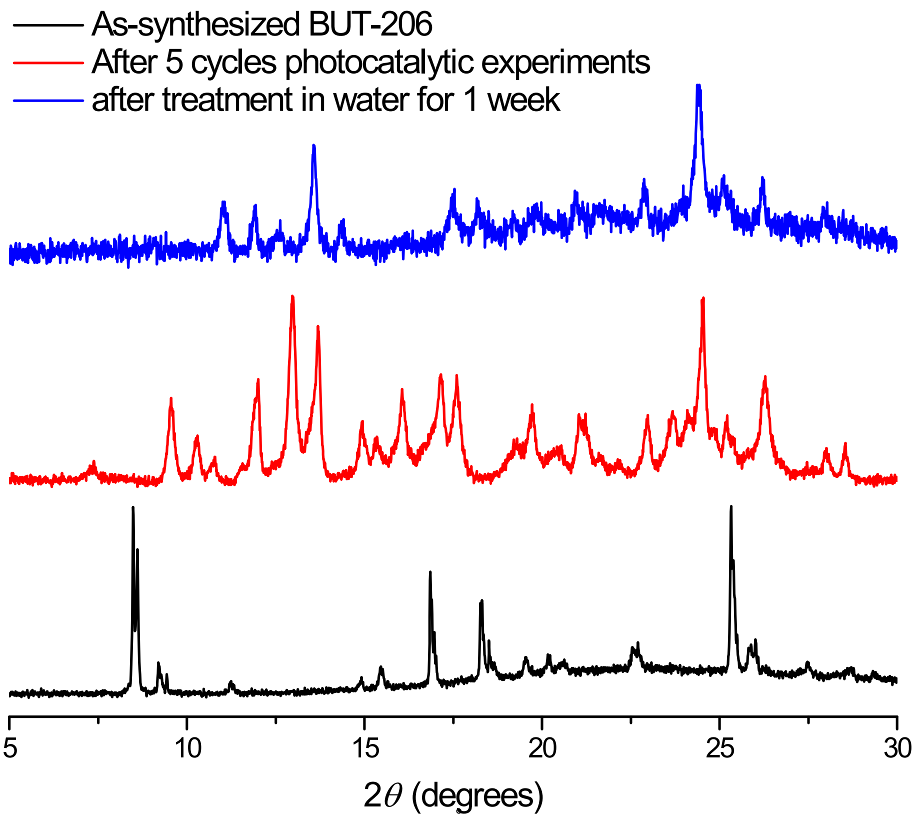

Fig. S6 XRD patterns of as-synthesized sample of **BUT-206** (black), the sample after 5 cycles experiments for photocatalytic degradation of crystal violet (red), and the sample after treatment in water for about 1 week (blue). The results indicated that the crystal structure of **BUT-206** significantly changed in water and in the photocatalytic reactions, although the photocatalytic activity of the sample mostly retained during the experiments.

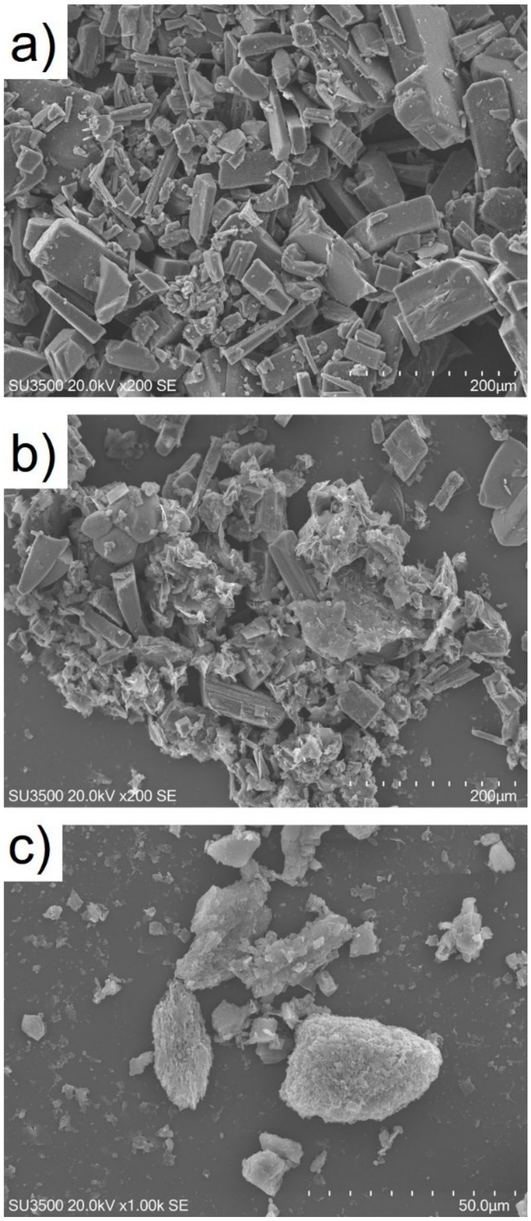

Fig. S7 SEM imgaes of as-synthesized sample of **BUT-206** (a), the sample after 5 cycles experiments for photocatalytic degradation of crystal violet (b), and the sample after treatment in water for about 1 week (c).

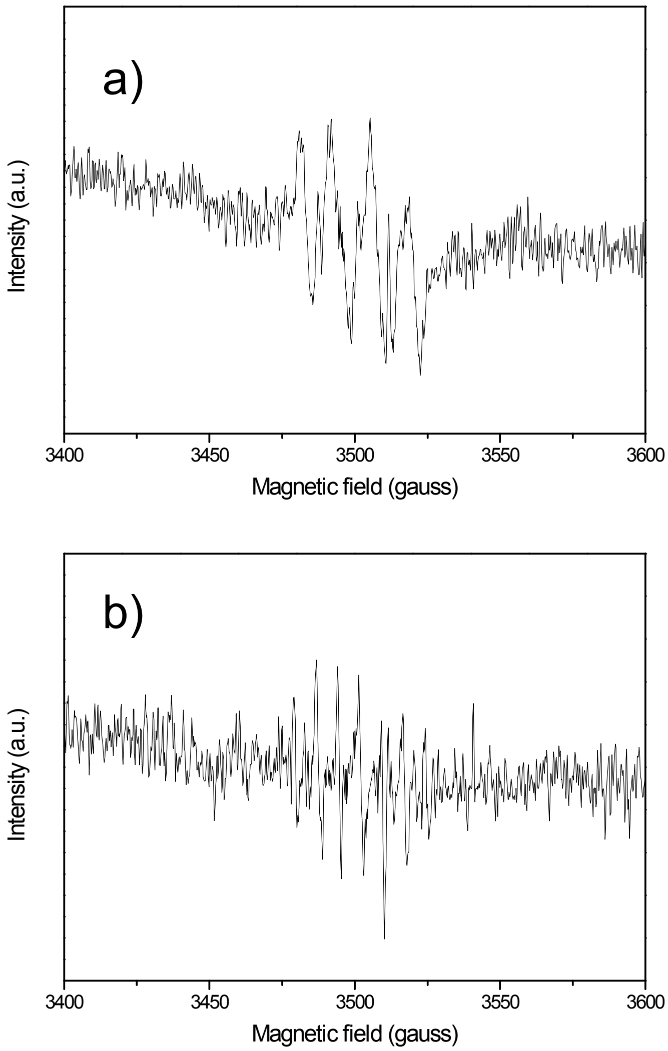

Fig. S8 ESR spectra of superoxide anion radicals (a) and hydroxyl radicals (b) formed in the photocatalytic reactions with BUT-206 as catalyst.

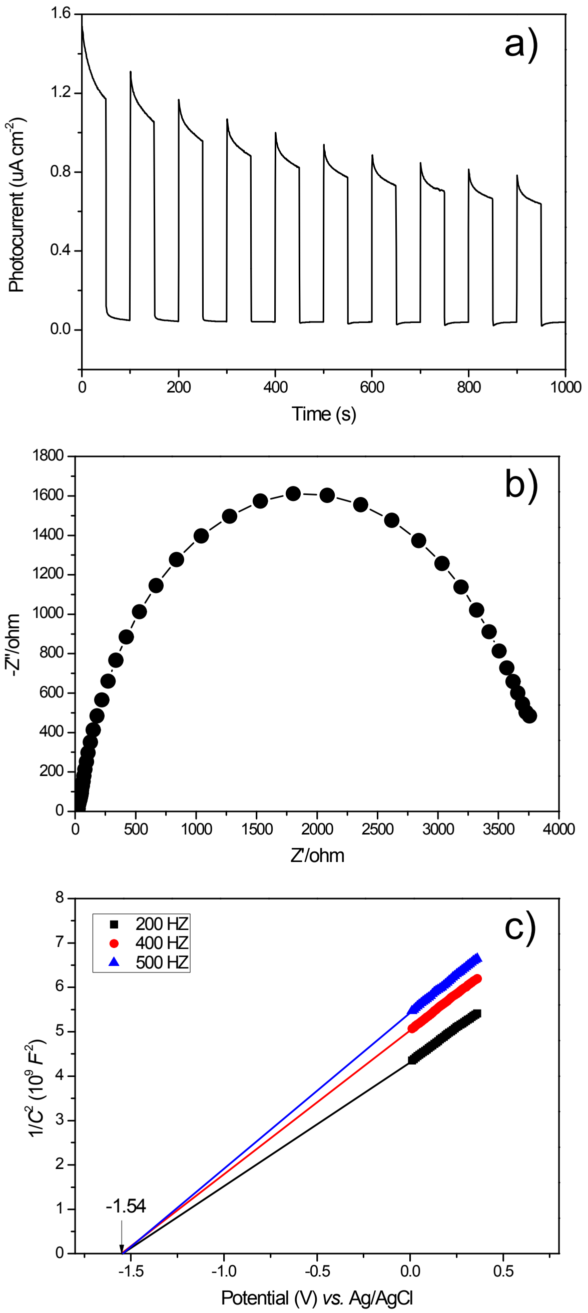

Fig. S9 (a) photocurrent responses, (b) EIS Nyquist plot, and (c) Mott-Schottky curves of BUT-206.

Table S1. Crystal and structural refinement data for **BUT-206**

Formula	$C_4H_7N_{15}O_2Zn_2$
Formula weight	428.03
Space group	*P*2/*c*
Crystal system	Monoclinic
a (Å)	9.9904(6)
b(Å)	6.0482(5)
c (Å)	21.616(2)
β (°)	101.289(8)
V (Å^3)	1280.85(18)
Z	2
Density calculated (g cm^{-3})	1.51952
Goodness-of-fit on F^2	1.005
F(000)	568
Absorption co-efficient (mm^{-1})	1.762
R_{int}	0.0737
R_1/wR_2 (all Data)	0.1075/0.1991
Theta range for data collection(°)	4.171 to 65.026.
Data / restraints / parameters	2187/142/164
Completeness to theta = 65.026°	100%

Final *R* indices [*I*>2sigma(*I*)]	R_1=0.0711, wR_2=0.1710
R indices (all data)	R_1=0.1008, wR_2=0.1948
Absorption correction	None
Refinement method	Full-matrix least-squares on F^2
Index ranges	-11<=h<=9, -7<=k<=5,-25<=l<=23

Table S2. Expected intermediates from the degradation of crystal violet by **BUT-206**.

Intermediates	Molecular structures
Ethane-1,2-diol	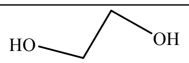
2-Hydroxypropanoic acid	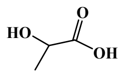
2,6-Dimethylbenzoic acid	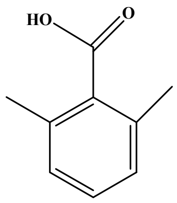
Piperidine-2-carboxylic acid	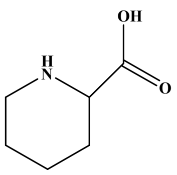

Benzoic acid	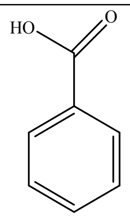
Pyrocatechol	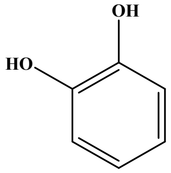
Adipic acid	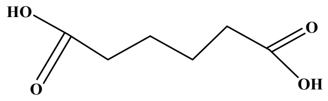
